# Surgical management of pleural empyema in the very elderly

**DOI:** 10.1308/003588412X13171221592212

**Published:** 2012-07

**Authors:** M Schweigert, N Solymosi, A Dubecz, M Beron, L Thumfart, D Oefner-Velano, HJ Stein

**Affiliations:** ^1^Klinikum Nürnberg Nord,Germany; ^2^Szent István University, Budapest,Hungary; ^3^Paracelsus Medical University, Salzburg,Austria

**Keywords:** Pleural empyema, Pneumonia, Very elderly patients, Sepsis

## Abstract

**INTRODUCTION:**

Parapneumonic pleural empyema is a critical illness. Age is an acknowledged risk factor for both pneumonia and pleural empyema. Furthermore, elderly patients often have severe co-morbidity. In the case of pleural empyema, their clinical condition is likely to deteriorate fast, resulting in life threatening septic disease. To prevent this disastrous situation we adapted early surgical debridement as the primary treatment option even in very elderly patients. This study shows the outcome of surgically managed patients with pleural empyema who are 80 years or older.

**METHODS:**

The outcomes of 222 consecutive patients who received surgical therapy for parapneumonic pleural empyema at a German tertiary referral hospital between 2006 and 2010 were reviewed in a retrospective case study. Patients older than 80 years were identified.

**RESULTS:**

There were 159 male and 63 female patients. The mean age was 60.5 years and the overall in-hospital mortality rate was 7%. Of the 222 patients, 37 were 80 years or older (range: 80–95 years). The frequencies of predominantly cardiac co-morbidity and high ASA (American Society of Anesthesiologists) grades were significantly higher for very elderly patients (*p*<0.001). A minimally invasive approach was feasible in 34 cases (92%). Of the 37 patients aged over 80, 36 recovered while one died from severe sepsis (in-hospital mortality 3%). There was no significant difference in mortality between the very elderly and the younger sufferers (*p*=0.476).

**CONCLUSIONS:**

Early surgical treatment of parapneumonic pleural empyema shows excellent results even in very elderly patients. Despite considerable co-morbidity and often delayed diagnosis, minimally invasive surgery was feasible in 34 patients (92%). The in-hospital mortality of very elderly patients was low. It can therefore be concluded that advanced age is no contraindication for early surgical therapy.

Pneumonia is one of the most common inflammatory diseases worldwide. Parapneumonic pleural effusion advances not infrequently to life threatening empyema. Age is an acknowledged risk factor for both pneumonia and pleural empyema.^1,2^ However, the question of optimal management of very elderly patients sustaining parapneumonic pleural empyema is yet unanswered.^3^

On the one hand, the very elderly often have substantial co-morbidity. Functional inoperability is therefore often assumed and most octogenarians are managed conservatively. On the other hand, empyema treated conservatively or by simple chest tube insertion is associated with high mortality and frequent treatment failure.^4–6^ Hence, the very elderly are disadvantaged by pre-existing illness as well as by suboptimal treatment.

Furthermore, their clinical condition is likely to deteriorate quickly while the prevention of devastating septic disease seems crucial to achieve a cure in the case of very elderly sufferers burdened by severe co-morbidity and poor performance status. Here, early surgical debridement using minimally invasive techniques might provide a practical solution. Video assisted thoracoscopic surgery has shown reliable good outcomes in the management of pleural empyema in several series in recent decades.^7–10^

Prompted by these encouraging results, we adopted minimally invasive surgical debridement of pleural empyema as the primary treatment option even for very elderly patients. No study exists to date regarding the outcome of surgical management of pleural empyema in octogenarians and nonagenarians. The aim of our study was therefore to particularly outline the results of operatively managed patients with pleural empyema who are 80 years or older.

## Methods

The study comprised 222 consecutive patients who underwent surgery for parapneumonic pleural empyema between January 2006 and December 2010 at the Department of General and Thoracic Surgery of the Klinikum Nürnberg Nord, a German tertiary referral hospital. The mean patient age was 60.4 years and there were 159 male (71%) and 63 female (29%) patients (Table 1). Only cases of primary parapneumonic empyema were included while empyema secondary to thoracic surgery was generally excluded. Moreover, cases of malignant pleural effusion or carcinosis pleurae were also excluded from the series. A local ethics committee approved this retrospective study.

**Table 1:** Characteristics of the complete series (*n*=222)

Men	159 (71%)
Women	63 (29%)
Mean age	60.5 years
Age range	17–95 years
Age <80 years	185 (83%)
Age ≥80 years	37 (17%)
Cardiac co-morbidity	90 (41%)
Pulmonary sepsis	46 (21%)
Empyema stage I	19 (9%)
Empyema stage II	146 (66%)
Empyema stage III	57 (26%)
In-hospital mortality	15 (7%)

The medical records of all patients participating in the study were analysed retrospectively. Information regarding demographic data, co-morbidity, ASA (American Society of Anesthesiologists)****grade, operative procedure, post-operative course and outcome was collected for each case. Cardiac co-morbidity was defined as the presence of congestive heart failure, cardiac valvular disorders, cardiovascular disease, arrhythmia or prior cardiac surgery. The whole study population was divided by age into two subgroups. The first subgroup was formed of patients younger than 80 years (‘younger patients’) and the second subgroup included all patients 80 years or older (‘very elderly patients’). Differences in pre-existing co-morbidity, ASA grade, occurrence of sepsis and outcome were analysed.

### Diagnostic procedure

Pre-operatively, all patients received ultrasonography with thoracentesis. If the pleural fluid sample was either frank pus or showed a pH of less than 7.2 or lactate dehydrogenase levels above 1,000iu/ml, pleural empyema was assumed. Furthermore, computed tomography of the chest was obtained routinely for all patients on referral to the thoracic surgery department. Diagnosis of pleural empyema always depended on a combination of clinical, microbiological, laboratory and radiological findings. In accordance with the triphasic nature of pleural empyema and the common international classification, three stages of disease were established and each patient was differentiated into one of these categories. Stage I consisted of the first, exudative phase, stage II resembled the fibrinopurulent phase and stage III was defined as the organising phase when fibrin and suppuration are replaced by granulation tissue and empyema becomes a chronic disease.

### Surgical technique

All patients received general anaesthesia with a double lumen endotracheal tube for one lung ventilation. A left-sided double lumen tube was mainly used because of easier placement in the left principle bronchus. In cases without previous examination of the airways, bronchoscopy was performed via the endotracheal tube to rule out airway injury or obstruction. All patients were placed in the lateral decubitus position.

Video assisted thoracoscopy was performed using standard equipment. Two 10mm trocars were inserted in the same intercostal space. Detritus and retained fluid collections were removed under direct visual examination by the thoracoscope and the inflammatory peel was thoroughly separated from the visceral pleura by blunt and sharp dissection. Microbiological samples were routinely obtained. After removal of all liquid and viscous suppuration including fibrinous mass and pleural coating, the thoracic cavity was irrigated with Ringer’s solution.

Open decortications required either a posterolateral or muscle sparing anterior thoracotomy using a rib retractor. Following careful debridement, the whole pleural surface was decorticated and the fibrinous coating removed until complete re-expansion of the lung had been achieved. Chest tubes were inserted in all patients regardless of the operative procedure. We aimed to re-establish spontaneous breathing as soon as possible. Most patients were therefore extubated in the operating theatre.

### Post-operative care

After successful thoracoscopic or open decortication, all patients received sufficient analgesic administration as well as intravenous antibiotics and intense physiotherapy including positive airway pressure inhalation and mobilisation as early as feasible. In addition, many elderly patients received hyperalimentation to improve their nutritional status. Irrigation of the infected pleural cavity via the inserted chest tubes was performed twice daily using Ringer’s solution. If appropriate, the patients were treated initially at the intensive care unit (ICU).

### Statistics

Statistical analysis was performed using R statistical software (R Foundation for Statistical Computing, Vienna, Austria). The independence of the studied variable pairs was tested with Fisher’s exact test.

## Results

**Figure 1:**
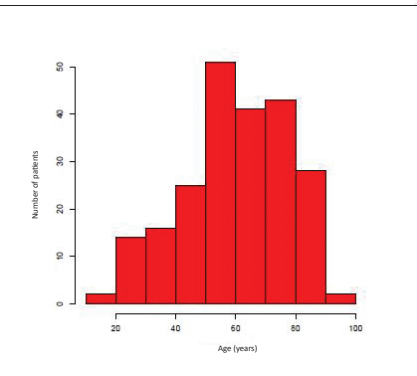
Age distribution in the study population

During the study period of 5 years, 222 patients were included in the series (Table 1). Most sufferers presented in the fibrinopurulent phase of empyema (stage II). Minimally invasive video assisted thoracoscopic decortication was performed in 199 cases (90%) while open decortication was mandatory in 23 patients (10%). The mean age was 60.4 years (Fig 1) and the mean ASA grade was 2.8, with a predominance of ASA grade 3 (Table 1). Cardiac co-morbidity was present in 90 cases (Table 1). Overall, 46 patients sustained sepsis and the general in-hospital mortality rate reached 7% (15/222). In total, 185 patients were younger than 80 years. For these patients, a primary minimally invasive approach was feasible in 89% (165/185) and the in-hospital mortality rate was 8% (14/185).

### Characteristics of very elderly patients

There were 37 patients (23 men and 14 women) who were 80 years or older (Table 2). The octogenarians generally suffered from severe, predominantly cardiac co-morbidity (Table 2). Moreover, diabetes mellitus and poor nutritional status were encountered frequently. Initial laboratory findings demonstrated raised a white blood cell count with average leucocytosis of 15.9 x 10^9^/l and an elevated mean C-reactive protein level of 18.0mg/dl. Furthermore, very elderly patients mainly suffered from mild anaemia with a median haemoglobin level of 11.1g/dl at hospital admission or referral to the department of thoracic surgery. The mean ASA grade was 3.2 and 8/37 patients (21%) sustained pulmonary sepsis.

**Table 2:** Characteristics of the very elderly patients (*n*=37)

Men	23 (62%)
Women	14 (38%)
Mean age	83.3 years
Age range	80–95 years
Mean ASA grade	3.2
Empyema stage I	1 (3%)
Empyema stage II	26 (70%)
Empyema stage III	10 (27%)
*Co-morbidity*	
Congestive heart failure	25 (67%)
Cardiac arrhythmia	14 (38%)
Cardiovalvular disorder	11 (30%)
Cardiovascular disease	6 (16%)
No cardiac co-morbidity	4 (11%)
Diabetes mellitus type 2	8 (22%)

A minimally invasive approach was initially feasible in 34 cases (92%). Treatment at the ICU was mandatory in 16 cases (43%) with a mean stay of 8.6 days. Persisting empyema required reoperation in six cases. While redoing of a minimally invasive procedure was feasible in two cases, three patients received an open window thoracotomy and one open decortication. In total, 36 patients recovered while 1 died, resulting in an in-hospital mortality rate of 3% for the very elderly (Table 2).

### Comparison between younger and very elderly patients

The ASA grade frequencies were distributed very unevenly between the age groups (Table 3). We therefore assumed that older patients had significantly higher ASA grades than patients younger than 80 years. Analysing the independence of ASA grade frequencies in the two age groups showed that it is very unlikely (Fisher’s exact test, *p*<0.001) that the ASA grades were independent from the age categories.

**Table 3:** Frequency of ASA (American Society of Anesthesiologists) grades in the age categories

	ASA grade 1	ASA grade 2	ASA grade 3	ASA grade 4	Low ASA grades (1/2)	High ASA grades (3/)4
Age ≥80 years	0	1	26	10	1 (3%)	36 (97%)
Age <80 years	4	63	108	10	67 (36%)	118 (64%)
Significance					***p*<0.001**	***p*<0.001**

**Figure 2:**
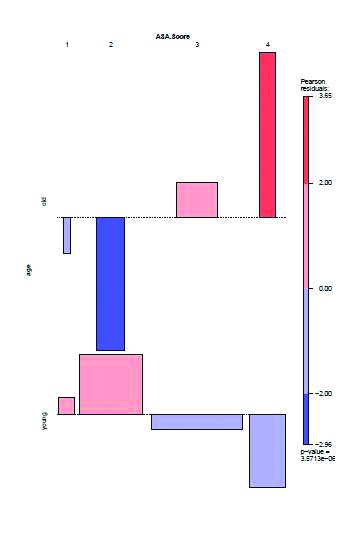
Association plot of age categories and ASA grades showing that among the older patients, ASA grades 3 and 4 are much more frequent and lower ASA grades (1, 2) are much more infrequent than would be expected in the case of independence. The younger patients show an opposite trend.

Looking at the association plot in Figure 2, it can be concluded that among older patients the higher ASA grades (grades 3 and 4) are much more frequent than is to be expected in the case of independence. Moreover, the lower ASA grades (grades 1 and 2) occur among the very elderly much less frequently than would be expected in the case of independence. The younger patients show just the opposite trend. We therefore divided the ASA grades into two categories (low and high) and tested the independence again with Fisher’s exact test (Table 3). This showed that for very elderly patients the odds of having higher ASA grades (ie grades 3 or 4) were 20 times higher than for the younger patients (odds ratio [OR]: 20.28, 95% confidence interval [CI]: 3.25–838.48, *p*<0.001).

Furthermore, the analysis showed that the proportion of patients with cardiac co-morbidity was rather different between the two groups. Only 57 of the younger patients presented with cardiac disorders while 128 had none. In marked contrast to this, 33 of the 37 very elderly patients suffered from pre-existing cardiac disorders. Fisher’s exact test demonstrated that the odds for cardiac co-morbidity among the very elderly were 18 times higher than those for the younger group (OR: 18.29, 95% CI: 6.10–74.33, *p*<0.001).

Pulmonary sepsis occurred in 46 cases (8 of the very elderly and 38 from the younger group). There was no significant difference of sepsis odds between the two groups (OR: 1.07, 95% CI: 0.39–2.64, *p*=0.828). Nevertheless, it seemed that the odds of sepsis among the very elderly were slightly higher compared with the younger group.

Overall, 15 cases had a fatal outcome. While only one of the very elderly died, in-hospital mortality in the younger group was 14 out of 185. Statistically, we found no significant difference regarding mortality between the two groups (OR: 0.34, 95% CI: 0.01–2.38, *p*=0.476). However, on closer examination of the results, it seems that the odds of fatal outcome among the very elderly are lower than in the group of younger patients. Thus, we recognised a surprising trend towards lower mortality in the very elderly although we could not demonstrate statistical significance.

## Discussion

Pleural empyema is a critical illness that was already known to Hippocrates. Since then, considerable progress has been accomplished in the management of this disorder. Notwithstanding this favourable development, mortality and morbidity are still substantial.^5,6^ Moreover, there is ongoing controversy regarding the appropriate treatment.^6,11^ In historic series, surgery was associated with marked mortality and medical measures therefore gained ground. However, conservative means or simple chest tube insertion are of low efficacy in cases of parapneumonic empyema.^5,6,11,12^ Frequent treatment failure and a mortality rate of up to 20% are the consequence.^5^ These discouraging results imply that conservative management with simple chest drainage should not be considered for empyema beyond the first exudative stage. During the 1990s, enzymatic decortication by instillation of fibrinolytic agents was described in order to break down loculation in the fibrinopurulent phase of empyema. However, results remained inconclusive and enzymatic decortication is hence now mostly reserved for otherwise inoperable patients.^4,13,14^

The invention of video assisted thoracoscopic surgery has revolutionised the surgical management of parapneumonic pleural empyema. Several large series have convincingly demonstrated feasibility as well as reliable good results of thoracoscopic debridement or decortication.^7–10^ Nowadays, therefore, minimally invasive thoracoscopic procedures are generally recommended for treatment of empyema, especially in the fibrinopurulent second stage. Subsequently, open decortication is reserved increasingly for the third, often chronic stage of disease or for patients who present with instable septic disease or underlying pulmonary disorders requiring major anatomical lung resections.

Nevertheless, very elderly patients have been somewhat excluded from this favourable trend. In the daily clinical routine, octogenarians are often deemed unsuitable for thoracic surgery due to severe co-morbidity, cachexia or poor functional status. Hence, they are generally managed conservatively. However, it is quite common that very elderly patients present with advanced disease at hospital admission. In our series, we found only one octogenarian with stage I pleural empyema.

Very elderly patients are disadvantaged for several reasons. Their advanced age is an acknowledged risk factor for both pneumonia and pleural empyema.^1,2^ Moreover, substantial co-morbidity, poor nutritional status and immobility add further difficulties. In these cases inappropriate management may result in a situation where recovery is impossible. Assumed functional inoperability often hampers timely referral for thoracic surgery. However, time is crucial to achieving success by minimally invasive approaches. The aim of this retrospective study was therefore to outline the results of operative management of parapneumonic empyema in very elderly patients. The preferred approach was video assisted thoracoscopic surgery with either pleural debridement or early decortication in case of transition from stage II to stage III disease. Our data show that this minimally invasive management is not only feasible in most cases but also provides encouraging results with low mortality.

Despite severe and often multiple co-morbidities, there was no statistically significant difference in mortality between very elderly patients and patients younger than 80 years. Given the significantly higher ASA grades and the at least equal frequency of sepsis, this is a remarkable finding. In our study, age was not correlated with fatal outcome. On the contrary, very elderly patients showed a slight trend towards reduced mortality.

The incidence of sepsis, the frequency of ICU stay and the mean duration of 8.6 ICU days together with the fact that there were 6 mandatory reoperations underline the high levels of disease in the group of very elderly patients. Nevertheless, the outcome was promising. We therefore propose that all very elderly patients suffering from parapneumonic pleural empyema should be assessed by an experienced thoracic surgeon regarding operability. Assumed functional inoperability due to age or co-morbidity should not be a reason for principal exclusion from operative management. A further decrease of the overall mortality of pleural empyema might be achievable by consistent referral patients including the very elderly for surgical debridement in early stage disease.

## Conclusions

Advanced age is associated with significantly higher ASA grades and significantly more co-morbidity for cases of parapneumonic pleural empyema. However, it is not related to worse outcomes and our study demonstrated no significant difference in mortality between the very elderly and other patients. We therefore recommend that minimally invasive surgical management should be considered in all reasonable cases regardless of age. Functional inoperability should not be declared without consulting an experienced thoracic surgeon.
